# Yellow nail syndrome: a review

**DOI:** 10.1186/s13023-017-0594-4

**Published:** 2017-02-27

**Authors:** Stéphane Vignes, Robert Baran

**Affiliations:** 1 Department of Lymphology, Centre National de Référence des Maladies Vasculaires Rares (Lymphœdèmes primaires), Hôpital Cognacq-Jay, 15, rue Eugène-Millon, 75015 Paris, France; 2 Nail Disease Centre, 42, rue des Serbes, 06400 Cannes, France

**Keywords:** Yellow nail syndrome, Respiratory manifestations, Sinusitis, Lymphedema, Review

## Abstract

Yellow nail syndrome (YNS; OMIM 153300, ORPHA662) is a very rare disorder that almost always occurs after 50 years of age but a juvenile or familial form has also been observed. YNS is diagnosed based on a triad associating yellow nail discoloration, pulmonary manifestations (chronic cough, bronchiectasia, pleural effusion) and lower limb lymphedema. Chronic sinusitis is frequently associated with the triad. YNS etiology remains unknown but a role of lymphatic impairment is usually evoked. YNS is more frequently isolated but may be associated in rare cases with autoimmune diseases, other clinical manifestations implicating lymphatic functions or cancer and, hence, is also considered a paraneoplastic syndrome. YNS management is symptomatic and not codified. YNS can resolve spontaneously. Oral vitamin E alone or even better when associated with triazole antifungals may achieve partial or total disappearance of nail discoloration. Pleural effusion can be treated surgically, with decortication/pleurectomy or pleurodesis. Antibiotic prophylaxis is prescribed for bronchiectasia with chronic sputum production. Lymphedema treatment is based on low-stretch bandages and the wearing of elastic compression garments combined with skin care, exercises and, as needed, manual lymph drainage.

## Background

The first case of yellow nail syndrome (YNS; OMIM 153300, ORPHA662) was probably reported by Heller in 1927 [1], but Samman & White described the first series of patients who had yellow nails associated with lymphedema in 1964 [2]. That report included 13 patients (six men, seven women; age range at onset 25–65 years), all of whom had very slow measured nail growth associated with abnormal nail-plate discoloration, ranging from pale yellow to dark greenish, and frequent onycholysis. Eight of them had ankle edema; one patient each had facial edema or Milroy’s disease (familial form of primary lymphedema). Four patients’ limb lymphangiograms showed lymphatic abnormalities, such as tortuous, dilated or hypoplastic vessels, which the authors considered suggestive of lymphatic dysfunction or defective lymph drainage being responsible for YNS. In this review, we analyze the available literature on this subject, describing clinical characteristics, explorations, associated diseases and management of this rare syndrome.

## Methodology

The literature search of the PubMed database used the words “yellow nail syndrome” for articles written in English or French. Other references cited in the identified articles were also considered.

## Definition

YNS is characterized by a triad of thickened yellow nails, primary lymphedema and respiratory manifestations. It is an acquired condition of unknown etiology. It is a syndrome – not a disease – that is associated with conditions as different as diseases implicating the lymphatic system, autoimmune diseases or cancers. Whereas Samman & White’s first description of YNS included only nail discoloration, Emerson added pleural effusion to the diagnostic criteria [3]. Among the three clinical YNS characteristics (yellow nail syndrome, respiratory tract involvement, lymphedema), only two are required to diagnose YNS but it is difficult to call the entity YNS without nail abnormality [4]. Moreover, the three components are not necessarily present simultaneously, and may appear individually and sequentially, thereby making YNS diagnosis difficult. The complete triad is present only in 27–60% of the patients [5–10] (Table 1). The percentage differences of a given clinical manifestation may be attributed to the medical specialty that recruited the patients.

**Table 1:** YNS clinical manifestations found in six large series of patients

Manifestation	Maldonado et al. [6], *N* = 41	Hoque et al. [5], *N* = 11	Piraccini et al. [7], *N* = 21	Nordkild et al. [8], *N* = 97	Varney et al. [9], *N* = 17	Pavlidakey et al. [10], *N* = 62
Yellow nails, *n* (%)	41 (100)	10 (91)	21 (100)	86 (89)	17 (100)	53 (85)
Chronic pulmonary manifestations, *n* (%)	23 (56)	7 (64)	15 (71)	61 (63)	17 (100)	24 (39) (PEs only)
Lymphedema, *n* (%)	26 (63)	6 (55)	6 (29)	78 (80)	13 (76)	45 (72)
Sinusitis, *n* (%)	17 (41)	3 (27)	3 (14)	NR	14 (83)	11 (18)
Complete triad	~60%	27%	29%	NR	76%	27%

*PEs* pleural effusions, *NR* not reported

## Epidemiology

No precise data are available to determine the exact prevalence of YNS, as fewer than 400 cases have been published in the literature, with an estimated prevalence <1/1,000,000. Cases have been described in all countries worldwide. YNS most often occurs in adults over 50 years old, with no sex predominance [5–7]. Pediatric forms are very rarely reported [11–21]: YNS may be present at birth (congenital) or develop before the age of 10 years [8].

A familial form of YNS has very rarely been described [5, 22–24], affecting two siblings [25, 26] or a family with eight cases in four sibships over two generations [22]. The very few reported familial cases mimic a dominant inheritance pattern, which is not supported by any genetic evidence [5]. YNS may be associated with intellectual disability, in which case it evokes a more complex syndrome [25] or occurs in cases of consanguinity [17].

## Diagnosis and diagnostic methods

### Yellow nails

**Fig. 1:**
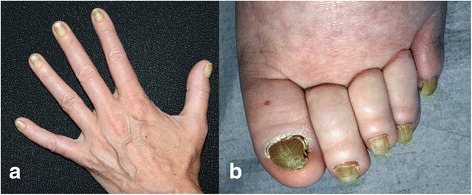
Yellowing of all 10 (**a**) finger and (**b**) toe nails

Yellow nails are the main clinical manifestation leading to YNS diagnosis. However, the possible interval between the first clinical sign (lymphedema, lung manifestations) and nail discoloration hinders affirmation of the YNS diagnosis. That yellowing represents a subset of chromonychia, defined as pathological nail discoloration, especially xanthonychia (yellow nail coloration). Nail discoloration varies from pale yellow to more or less dark greenish [27]. The nail plate becomes thickened, with an enhanced transverse curvature (overcurvature), sometimes with a notable hump, cross-ridging, very hard (scleronychia) and difficult-to-trim nail, and cuticle disappearance [28]. Usually opaque, the lunula disappears because of nail hyperkeratosis [27] (Fig. 1). Erythema may be seen in the proximal nail fold, frequently associated with chronic paronychia). Onycholysis (distal nail plate–nail bed separation) may occur with possible proximal spreading, leading to complete nail shedding [29, 30]. Longitudinal growth of the affected nail (0.23 mm per week) was half that of a normal nail (0.46 mm per week) [2, 31]. The affected nail’s thickness (0.97 mm) was double that of a normal nail (0.57 mm), suggesting that the nail that grows half as fast and twice as thick [31].

### Pulmonary manifestations

Lung involvement in YNS, which occurred in 56–71% of the patients, diversely affected some parts of the respiratory tract with a variety of clinical manifestations [6–8]. Chronic cough is the most frequent pulmonary manifestation seen in 56% of YNS patients [6], with pleural effusions found in 14–46% of the patients [6, 7].

Based on their retrospective systematic review of more than 150 patients described in publications identified with the search terms “pleural effusion” and “YNS”, Valdés et al. recently reported the characteristics of these pleural effusions [32]: 68.3% were bilateral; the fluid appeared serous in 75%, milky (chylothorax) in 22% and purulent (empyemas) in 3.5%; 95% of effusions were described as exudates (median protein level: 4.2 g/dl) and 5% as transudates that harbored a median nucleated cell count of 1540 cells/mm^3^ with 96% lymphocytic predominance.

However, sputum bacteria (*Pseudomonas aeruginosa*, *Haemophilus influenzae*, *Streptococcus pneumoniae*, *Moraxella catarrhalis*) are the same in idiopathic and YNS-associated bronchiectasias [33]. Recurrent pneumonias occur in 22% of the patients. Also, bilateral apical fibrosis, patchy alveolar infiltrates and cystic lesions are very rarely observed in YNS patients [33, 34].

YNS patients’ pulmonary function test results are usually normal or may indicate a moderate-to-severe restrictive syndrome attributable to pleural effusions [4]. Extremely rare patients may have mixed obstructive–restrictive syndrome or decreased diffusion capacity [6]. Histological examination of pleural biopsies revealed normal morphology or that of chronic fibrosing pleuritis, and did not provide any further information; biopsies are usually not contributive [32]. Bronchiectasias are present in 44%. Chest computed-tomography (CT) scan is the best imaging technique to diagnose bronchiectasia, which, in YNS patients, is significantly less extensive, severe and with lower bronchial wall thickness scores than in matched idiopathic bronchiectasia patients [33].

### Lymphedema

**Fig. 2:**
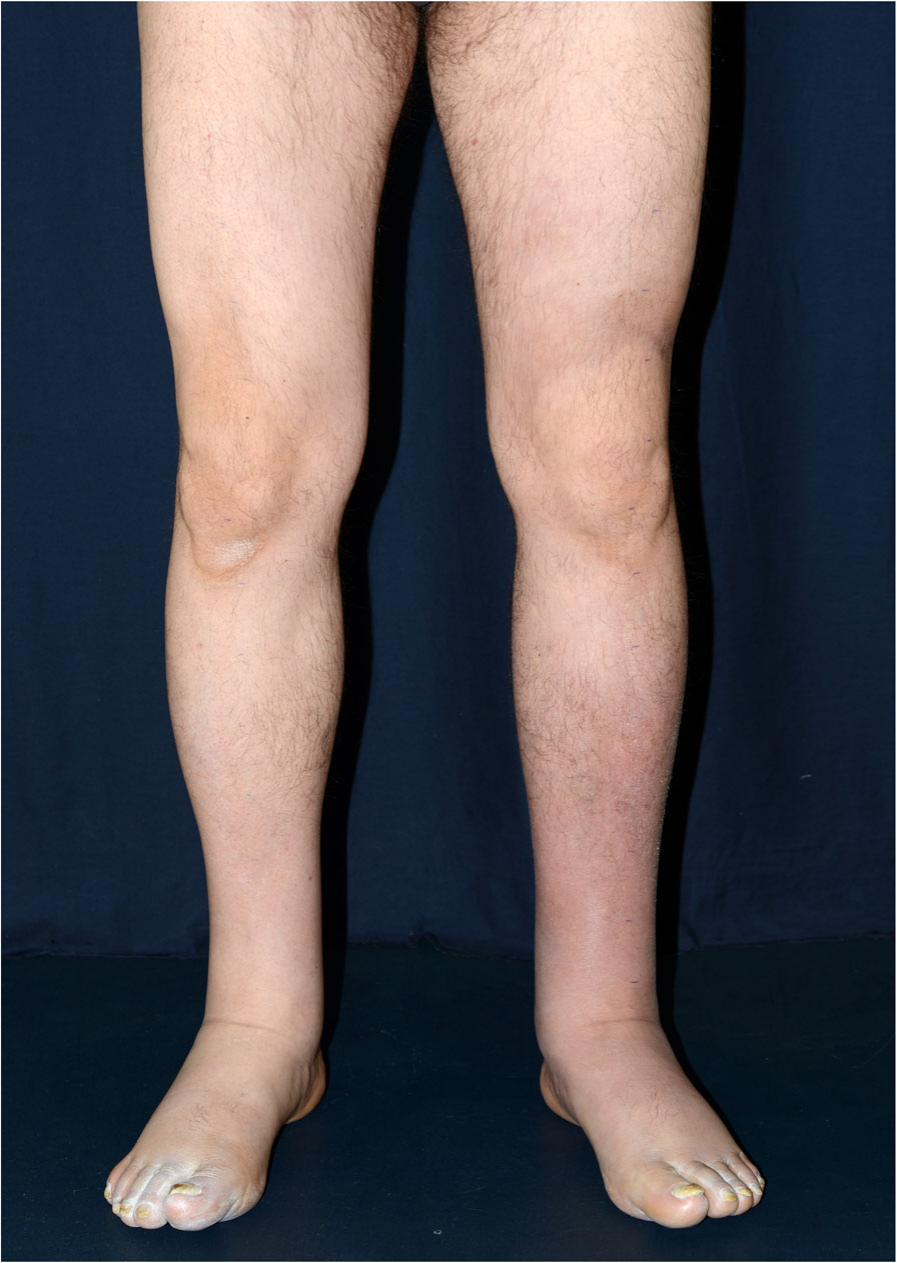
Bilateral lower limb lymphedema involving the feet, ankles and calves, with accentuation of the flexion folds

Lymphedema is a clinical feature of YNS, occurring in 29–80% of the reported series, and may be the first sign of the disease in about one-third of them [6–8]. Lymphedema characteristics do not differ from those of primary lymphedema. It involves the lower limbs, especially bilateral and below the knee (Fig. 2). The increased volume of the lymphedematous limb is caused by excess lymph accumulation but also fibrosis resulting from fibroblast stimulation and excess adipose tissue due to adipocyte stimulation [35, 36]. Stemmer’s sign (inability to pinch the skin on the dorsal side or the base of the second toe) is pathognomonic of lymphedema and is fibrosis-related. Superficial edema is responsible for the more-or-less present pitting edema. Lymphedema is a chronic disease, with a major tissular component leading to incomplete reversibility under treatment. Although cellulitis (erysipelas) is the main lymphedema complication, discomfort, esthetic prejudice and diminished quality of life also complicate the disease [37, 38].

### Sinusitis

**Fig. 3:**
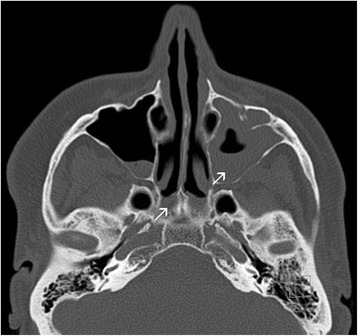
Sinus computed-tomography scan: note the subtotal opacity of the left maxillary sinus and ethmoidal sinusitis

Acute or chronic rhinosinusitis is very common in YNS patients, estimated between 14 and 83% [5–10]. The maxillary sinus is the most frequently affected, followed by ethmoid, frontal and sphenoid [9] (Fig. 3). Nasal symptom onset may precede nail changes by a few years, appear simultaneously or arise subsequently. Clinical signs include daily mucopurulent rhinorrhea, nasal obstruction and frequent post-nasal drip. Nasal airway examination usually finds narrowed nasal pathways, mucosal inflammation with variable enlargement of the turbinates and the presence of mucopus. Other symptoms may be associated, e.g., headaches or recurrent facial pain. Non-contrast sinus CT scans show mucosal thickening, with fluid levels sometimes reported.

### Other manifestations

Very rare ocular involvement has been reported: chemosis, corneal micropannus (vascularized sheet of fibrous tissue overlying the cornea), eyelid lymphedema, thickened conjunctiva [39, 40]. Anecdotal associations have also been described: anhydrosis, pectus excavatum, eosinophilia–myalgia syndrome, bullous stomatitis, sarcoidosis and Raynaud’s phenomenon, cerebral aneurysm and pancytopenia [6].

### Children

Among children with YNS, 75% had lung manifestations (infections, pleural effusions, bronchial dilations and/or bronchial cysts) and ear-nose-throat symptoms in 31%, with a moderate female predominance [20]. Lymphedema prevalence ranged from 56 to 80% of YNS children and may appear later than the nail discoloration [8].

## Pathogenesis

**Fig. 4:**
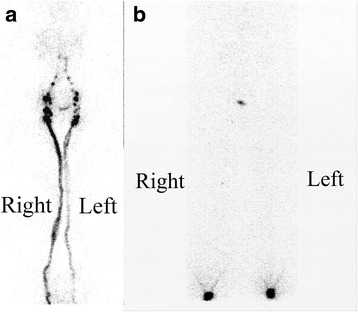
Lower-limb lymphoscintigraphy images were obtained 40 min after injecting ^99m^technetium-labeled colloidal albumin into two patients with the complete YNS triad: moderate lymphostasis and slightly decreased (**a**) or absent (**b**) inguinal lymph-node uptake

Although YNS etiology of remains unknown, some hypotheses were advanced. Lymphatic involvement is often evoked to explain lymphedema, pleural effusion (particularly chylothorax) or nail discoloration but it is difficult to implicate it in bronchiectasia and sinusitis. Lymphatic impairment is not easy to confirm. Four YNS patients underwent lower limb direct lymphangiography, less used at present, but lymphatic abnormalities were noted only in the patient with severe lymphedema. Quantitative limb lymphoscintigraphy with ^99m^Tc-colloidal antimony sulfide revealed less activity (percentage uptake) in the draining lymph nodes (inguinofemoral or axillary) [41]. Moreover, the uptake percentages in the axillary/inguinal lymph nodes of the YNS group were significantly lower than those of the normal controls but significantly higher than those of subjects with primary or secondary lymphedema, hence more suggestive of impaired lymph transport than the lymphatic hypoplasia/aplasia seen in true primary lymphedema. Furthermore, the YNS group without lower limb edema had better lymphatic drainage than those with edema [42] (Fig. 4). Maldonado et al. thought that YNS pathophysiology might be attributable to microvasculopathy associated with protein leakage rather than functional lymphatic impairment [43]. Notably, nailfold capillaroscopy occasionally showed dilated and tortuous capillary loops [44].

Defective lymphatic drainage might be responsible for the slow growth and thickened nails observed in YNS, and may reflect subungual tissue sclerosis leading to lymphatic obstruction. Light microscopy examination of sections of nail-matrix tissue revealed replacement of the normally loose fibrovascular subungual stroma by dense, fibrous tissue (composed of dense collagen deposits) extending from the immediate subepithelial stroma to a depth of 2.5 mm. Numerous ectatic, endothelium-lined channels were prominent within the fibrotic stroma [45]. Fibrosis and dilated lymphatic vessels were also seen in the parietal pleura of a YNS patient [46, 47]. The accumulation of lipofuscin pigment was advanced to explain the yellow discoloration [48], whereas abnormal nail keratinization might be explained by the presence electron microscopy-visualized keratohyalin granules, not found in normal adult nails.

More recently, it was hypothesized that titanium, especially titanium dioxide, might play a role in YNS. High titanium levels (determined by energy dispersive X-ray fluorescence) were detected in the nails of YNS patients but not in control nails. The authors postulated that titanium ions were released from titanium implants (inlays, crown) in the teeth or jaws through the galvanic action of amalgam or localized oxidative action of fluorides [49–51]. Other sources of titanium ions were also suggested: joint implants, surgical staples, foods (chewing gum to try to explain YNS in children), medication excipients, cosmetics (sunscreen, moisturizers, shampoo, toothpaste) [50, 52]. Titanium’s hypothetical role remains possible, but probably not sufficient, because its presence in other organs (liver, spleen, lymph nodes, lung) of autopsied patients was not accompanied with nail yellowing [53].

## Associated diseases

Several infants had YNS associated with non-immune hydrops fetalis; this association is probably not fortuitous [54]. Non-immune hydrops fetalis was present at birth [20, 55]. A child with YNS had a brother who died of non-immune hydrops fetalis, suggesting a possible relationship between the two diseases [17].

YNS is very rarely associated with primary intestinal lymphangiectasia (Waldmann’s disease) (OMIM 152800, ORPHA90362) or lymphedema–distichiasis syndrome (OMIM 153400, ORPHA33001), suggesting that these entities have overlapping characteristics, including lymphatic impairment [56, 57]. Waldmann’s disease is characterized by primary intestinal lymphangiectasia, with lymph leakage into the bowel lumen leading to hypoalbuminemia, hypogammaglobulinemia and lymphopenia [58]. Distichiasis is defined as double or more rows of eyelashes localized on the Meibomian gland orifices [59].

The YNS association with malignant disease raises the hypothesis that it might be a paraneoplastic syndrome but that notion remains controversial. The frequency of cancer being diagnosed concurrently or closely thereafter in YNS patients was estimated at 4/41 [6] and 1/21 [7]. Various types of cancers were associated with YNS: bronchial carcinoma [60, 61], breast [7, 62, 63], non-Hodgkin lymphoma [64, 65], gallbladder [6, 66], larynx [67], renal cell carcinoma [6], endometrium [68], melanoma [3], multiple myeloma after hematopoietic stem-cell transplantation [69] or precancerous mycosis fungoides [28]. The YNS-to-cancer-diagnosis interval ranges from days to years, with gradual development of the complete YNS triad [61].

YNS was occasionally associated with autoimmune diseases [70], immunodeficiency disorders, such as common variable immunodeficiency, combined T- and B-cell deficiency [70, 71], Guillain–Barré syndrome [72], nephrotic syndrome [73, 74], Hashimoto’s thyroiditis, severe hypothyroidism or hyperthyroidism [75–77], xanthogranulomatous pyelonephritis [78] and rheumatoid arthritis even without thiol-analog use [79].

Immunological studies on YNS patients are very scarce. Isolated case reports associated YNS with IgA deficiency [22] or hypogammaglobulinemia [80]. Bokszczanin & Levinson described a 57-year-old woman with YNS and poor selective responses after vaccination against *Streptococcus pneumoniae* and *Haemophilus influenzae* [81], which might explain, in part, the recurrent lung or sinus infections in YNS. Gupta et al. reported lymphopenia in two YNS patients (one with common variable immunodeficiency) with low percentages of CD4^+^ T cells, high percentages of CD8^+^ T cells and severe naïve CD4^+^ and CD8^+^ T-cell deficits responsible for muted T-cell responses to antigens. A suggested mechanism for diminished naïve T-cell subsets might be less thymus output (thymus involution and/or apoptosis) [70]. It is of interest to note that, in another rare disease with lymphatic abnormality, primary intestinal lymphangiectasia (Waldmann’s disease), immunological investigation results were similar to those of YNS patients [82].

## Differential diagnosis of nail discoloration

### Drugs

d-Penicillamine, bucillamine and tiopronin are three thiol compounds used for long-term treatment of rheumatoid arthritis. For the rare cases of drug-related YNS, nail discoloration was the first manifestation in 88% of them, but it was less frequently associated with pleural effusion and lymphedema than in YNS not drug-related [83, 84]. Competitive inhibition of disulfide-binding in keratin biosynthesis is postulated to explain the major slowing of nail plate growth in bucillamine-treated patients. Moreover, thiol drugs contain cysteine, which is also a major nail component. After bucillamine withdrawal, nail discoloration declined in over 90% of the affected patients but lymphedema and pulmonary manifestations were attenuated in only 30–35% [84]. Gold and methotrexate, also used to treat rheumatoid arthritis, are less suspected of being associated with YNS [85].

### Infections

Nail yellowing is abnormal and may be attributable to something other than YNS. Nail infection or mycosis should be ruled out before considering YNS. *Candida-, Aspergillus-* or dermatophyte-caused nail mycosis may cause such discoloration. *Pseudomonas aeruginosa*, via production of the blue–green pigments pyoverdin and pyocyanin, may be responsible for chloronychia (green rather than yellow nail discoloration) in the elderly [86]. Chloronychia is more common in homemakers, barbers, dishwashers, bakers and medical personnel.

### Others

In children and adults, differential diagnoses include planus lichen, psoriasis or alopecia areata, chronic paronychia, onychogryphosis and acquired pachyonychia [87–89]. Yellow nail discoloration may also have rare local and toxic causes (Table 2) [90].

**Table 2:** Rare, usually work-related, local toxic causes of yellow nail discoloration, from [90]

Epoxy systems: metaphenylenediamine, 4,4′-methylenedianiline
Flower handling
Pesticides: diquat, paraquat, dinitroorthocresol, dinobuton
Chromium salts
Dyestuffs: dinitrosalicylic acid, dinitrobenzene, dinitrotoluene, trinitrotoluene

## Treatment

YNS treatment is not codified. YNS may resolve in few months without treatment [91] or, when it is a paraneoplastic syndrome, after cancer therapy [62].

### Yellow nail changes

The main aim is to improve the frequently unesthetic nail appearance and associated pain, due, in part, to onycholysis. A few drugs have been proposed to treat the nail discoloration with inconsistent efficacy. None of the following treatments can be recommended systematically to treat YNS.

#### Systemic treatments of yellow nails

Oral vitamin E is the only agent that successfully treated YNS [48, 92–95]. Oral α-tocopherol (vitamin E) was frequently prescribed at 1000–1200 IU/day, with incomplete or inconstant efficacy. Norton’s hypothesized, as follows, that vitamin E would be effective: lipofuscin pigments, possibly responsible for nail yellowing, are derived from colorless lipid precursors, transformed by oxidation in tissue to produce varying degrees of yellow; vitamin E has proven in vitro antioxidant properties, and in vivo might protect cell membranes against free-radical–mediated oxidative damage, thereby potentially blocking lipofuscin-pigment production [48].

Although YNS is not caused by fungal infection, triazole antifungals were regularly used to treat it. Itraconazole, given at 400 mg/day for 1 week/month for 6 months, achieved only two mild attenuations and two cures among eight patients (one relapsed after drug discontinuation) [96]. Among the 13 patients who took oral fluconazole (300 mg once weekly) and oral α-tocopherol (1000 IU/day), two benefited from clinical improvement and 11 were considered clinical cures [97], without any efficacy on other YNS manifestations. One of the hypotheses to explain that partial efficacy is based on azole antifungal stimulation of linear nail growth [98, 99].

Oral zinc sulfate supplementation (300 mg daily) obtained attenuation of nail yellowing or growth and lymphedema after 8 months of treatment but no modification of pulmonary manifestations [95].

Clarithromycin (400 mg/day, 6 years) successfully treated one patient [100].

A patient with common variable immunodeficiency treated with subcutaneous immunoglobulin mounted good responses in terms of frequency of infections, lymphedema and pleural effusions [70].

#### Local treatments

Intralesional steroids, such as topical triamcinolone acetonide (5 mg/ml/injection, 0.1–0.2 ml for each affected nail), were proposed alone or combined with fluconazole and vitamin E [92, 101].

In a first study published in 1991, Williams et al. prescribed topical vitamin E; the treated nails improved clinically and growth rates rose [94]. In a randomized study using a vitamin E preparation (solution of 20,000 IU of tocopherol acetate/fluid ounce of safflower oil) applied twice daily to the nails), no difference (appearance or nail growth) versus placebo was observed after 6 months of administration [23].

### Pulmonary manifestations

Symptomatic treatments are prescribed. Patients may receive antibiotics for acute exacerbation of bronchiectasia, whereas, for patients with poor symptom control and/or recurrent exacerbations, low-dose antibiotic prophylaxis, such as oral azithromycin (usually 250 mg 3 times/week), achieved attenuation of chest symptoms for the majority of them [33]. Physiotherapy training (postural drainage, chest physiotherapy, flutter valve), combined or not with antibiotic prophylaxis, is also prescribed to help patients self-manage their chronic expectoration.

Vaccinations against flu and pneumococci are strongly recommended. Surgical intervention of recurrent and/or large pleural effusions is useful: decortication/pleurectomy, pleurodesis (talc [47, 102], picibanil [103], quinacrine [4]) and pleural–peritoneal shunts were the most effective treatments of symptomatic pleural effusions with, respectively, 89, 82 and 67% partial or complete responses [33].

Octreotide, a somatostatin analog, was also used to treat YNS pleural effusions or chylous ascites and lymphedema, and generated positive responses [47, 104–107]. Somatostatin analogues reduce intestinal lipid absorption and lower the triglyceride concentration in the thoracic duct in animals [108]. Those actions could explain the diminution of the chylous but not non-chylous effusions present in most YNS patients. Octreotide was initially administered subcutaneously (0.5 mg twice daily) to ensure safety, followed by the long-acting repeatable formulation (30 mg given once/month) with or without progressive dose diminution [105, 107]. One initial octreotide responder became “resistant”, suggesting tachyphylaxis to long-lasting treatment, as previously described for acromegaly patients receiving chronic treatment. Lanreotide, an alternative somatostatin analog, may be useful for such cases [47, 109].

### Lymphedema

Complete decongestive therapy, also called complex or multimodal decongestive physiotherapy, is the term proposed by Michael Földi in the 1980s to define lymphedema treatment. This approach is divided into two separate phases [110]. The first, intended to obtain the most important lymphedema-volume reduction, is comprised of several components: low-stretch bandage, manual lymph drainage, skin/nail care (to detect and eliminate potential sites of entry for infection) and exercises, each having its own specific objective and role in limiting the impact of this disorder. The intensive strategy of this stage aims to achieve 30–40% lymphedema-volume reduction [111], eliminating only the fluid component of lymphedema. The second phase of complete decongestive therapy helps stabilize lymphedema volume over the long-term and is based on wearing a high-pressure elastic garment, exercises, skin care and, sometimes, manual lymph drainage [112]. Each patient should be offered several training sessions in validated specific patient-education programs to master the wrapping procedure and verify good understanding and implementation. Overnight bandaging at least three times per week is recommended during long-term maintenance. The aim of learning self-bandaging is to improve the patient’s autonomy to manage his/her own lymphedema [113].

### Sinusitis

Treatment of acute sinusitis is based on antibiotics (amoxicillin–clavulanate (1.5–3 g/day), or, in the case of penicillin allergy, doxycycline (200 mg/day), fluoroquinolone (levofloxacin, 500 mg/day) or moxifloxacin (400 mg/day)) for 5–7 days [114]. Treatment of chronic sinusitis is not specific for YNS patients but global responses to medications, including short-course oral antibiotics, topical intranasal steroids, saline irrigation and topical or oral decongestant, are poor [115]. Surgical procedures may be necessary and are essentially based on endoscopic sinus surgery (endoscopic middle meatal antrostomy, conventional inferior meatal antrostomy) [116].

## Prognosis

Spontaneous remission of the nail changes has been observed in up to 30% of the YNS patients, regardless of treatment [5]. Remission of nail changes was more likely for fingernails than toenails, perhaps because of persistent lower limb lymphedema, which might maintain the presumed lymphatic pathophysiology [5]. More generally, the attenuated discoloration is not associated with simultaneous regression of other systemic manifestations. In YNS associated with malignant disease, treatment of the latter may lead to attenuation or disappearance of the clinical YNS signs [62, 69]. In Maldonado et al.’s study, 17 of the 37 patients with available follow-up information died after a median of 82 months [6]. In that study, a Kaplan–Meier survival curve estimated median survival at 132 months, shorter than that of a paired-control population.

## Conclusion

YNS is very rare disorder associating yellow nail discoloration, lung manifestations/sinusitis and lymphedema. It is more frequently isolated but may be associated with other diseases implicating the lymphatic system, autoimmune diseases or cancers. Its etiology remains unknown, although lymphatic impairment is regularly evoked in the literature. Titanium is a more recent hypothetical agent but so far remains unconfirmed to explain the syndrome. YNS treatment is symptomatic for each component: yellow nails, pulmonary manifestations/sinusitis, lymphedema. Vitamin E combined with fluconazole, usually prescribed to treat yellow nails, achieves partial or complete responses. Spontaneous resolution is also possible. Research is required to better understand and treat this rare and very poorly recognized disease.

## Abbreviations

YNS: Yellow nail syndrome
